# Bilateral Ophthalmomyiasis Externa of Lid by Musca domestica: A Rare Presentation

**DOI:** 10.7759/cureus.60424

**Published:** 2024-05-16

**Authors:** Manjiri P Sune, Mona P Sune, Shital M Mahajan, Pradeep Sune

**Affiliations:** 1 Ophthalmology, Jawaharlal Nehru Medical College Wardha, Wardha, IND; 2 Ophthalmology, Sune Eye Hospital, Wardha, Wardha, IND; 3 Microbiology, Jawaharlal Nehru Medical College, Wardha, Wardha, IND

**Keywords:** oestrus ovis, musca domestica, conjunctivitis, blepharitis, myiasis

## Abstract

A case of severe blepharoconjunctivitis in the last three weeks diagnosed the slit lamp as external ophthalmomyiasis. On ocular examination, numerous pupae were present on the lid margins, firmly adhering to the lid lashes bilaterally. All of them were removed mechanically under topical anesthesia. They were 67 in number. Healing occurred without any complications. In such cases of blepharoconjunctivitis, physicians should consider the possibility of ophthalmomyiasis externa, especially in places where high numbers of livestock are found. Otherwise, there is a chance of missing the diagnosis, which can be met with a more serious condition called ophthalmomyiasis interna.

## Introduction

Myiasis is infestation by fly larvae or maggots [1]. Maximum cases affect the skin and rarely affect the eyes, paranasal sinuses, nasal passages [2], urinary and genital tract [3], and intestine [4]. Ocular involvement is seen only in less than 5% of cases [5,6]. Ocular involvement is mostly found in shepherds and farmers, mostly in rural areas, but it may also involve people without having these jobs. The most common causative agent is Oestrus ovis, and it presents in external and internal forms [5,6]. External ophthalmomyiasis mainly involves bulbar or palpebral conjunctiva. In internal ophthalmomyiasis, there is globe penetration by the larva. The external type is mostly self-limiting, but the internal form can result in globe destruction and severe vision loss. There can be an invasion of larvae in orbit, causing more severe destruction [7]. Ophthalmomyiasis is rare in India and common in Mediterranean countries. The aim of presenting this case of ophthalmomyiasis external with Oestrus ovis is to discuss the bilateral involvement of the lid and its treatment.

## Case presentation

**Figure 1:**
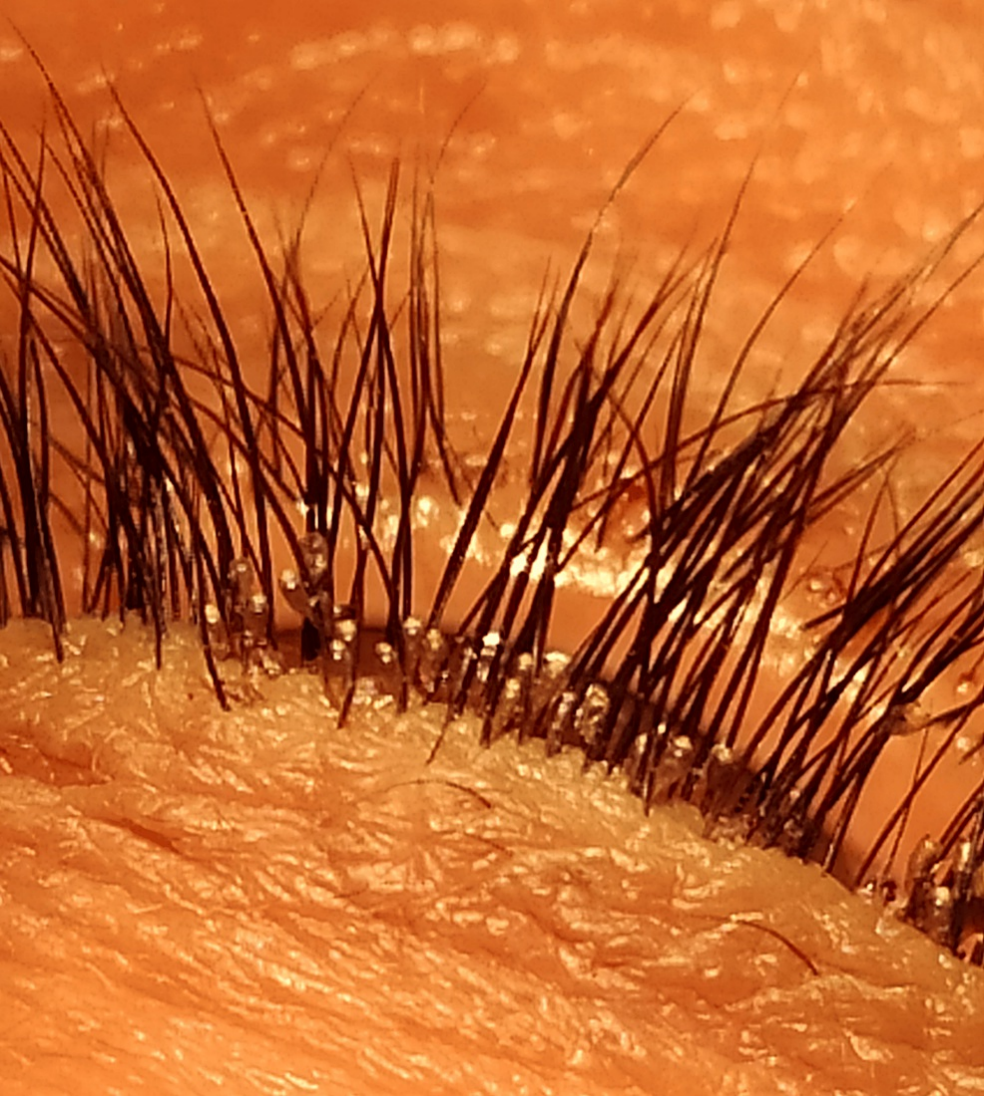
Pupae of Musca domestica adhered to lid margin along the eyelashes

A 49-year-old female patient presented to our clinic on April 22, 2020, with complaints of severe itching of the lids in both eyes, which started three weeks ago from severe redness and a foreign body sensation of the eyes, for which she took topical eye drops from a general practitioner. The patient was a housewife by occupation with no other systemic complaints. There is only a positive history of throwing garbage at a nearby garbage dump every day, where lots of flies could be seen. There was no history of contact with any animals. The redness decreased due to the topical eye drops, but the itching persisted. The patient could not visit an ophthalmologist as there is a period of lockdown due to COVID-19. The daughter's inspection revealed the presence of some motile organisms, which she removed at home using cotton buds and simple wiping techniques. The best corrected visual acuity (BCVA) in both eyes was 20/20. The slit lamp examination revealed bilaterally swollen lids with white scales and excoriation of skin on and around the lid margins. As per history, motile organisms were searched within fornices but no larvae were seen in the conjunctival sac. Still, there were numerous pupae present on the lid margins, firmly adhered to the lid lashes bilaterally, which were golden brown (Figure 1).

Management

Mechanical Removal

All of the pupae were removed mechanically under topical anesthesia (proparacaine 0.5%) with the help of plain forceps. The total number of pupae removed from both eyes was 67.

Medical Management

Following the procedure, the patient received topical flurbiprofen with Gatifloxacin (Flubigat, Entod, India) eye drops four times a day and Moxifloxacin eye ointment (Microgat, Micro Labs, India) at bedtime.

**Figure 2:**
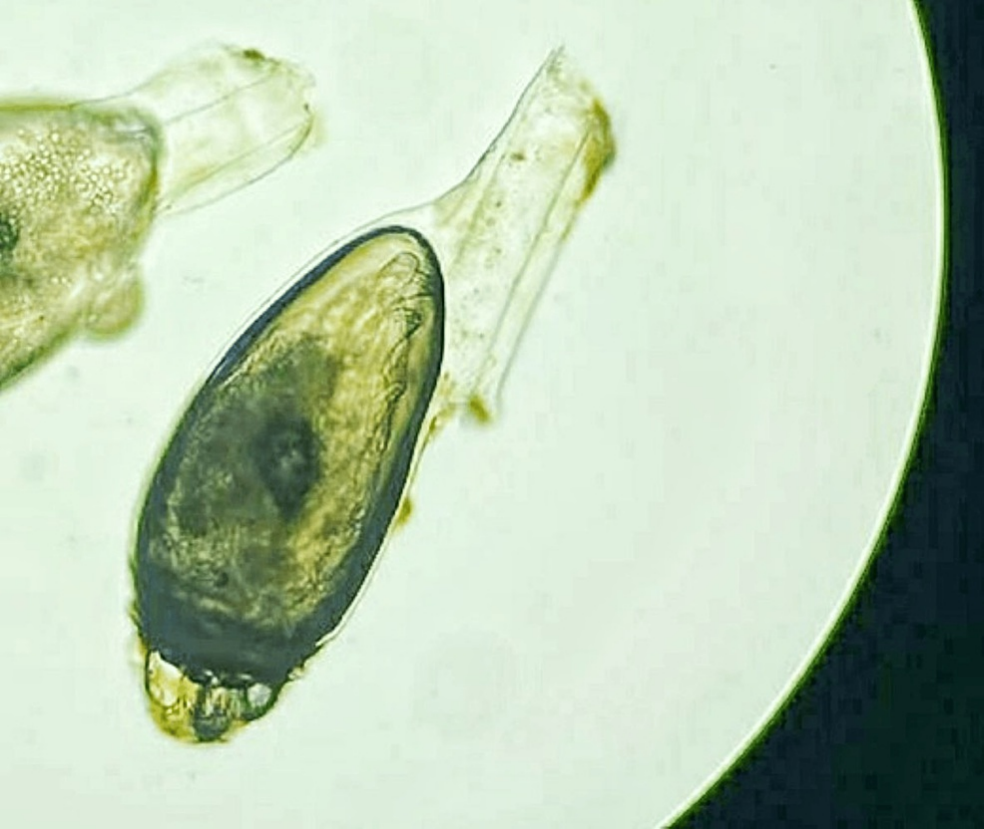
DSP (Dithiobis Succinimidyl Propionate) fixed slide showing pupae of Musca domestica with anterior part and posterior wing projections

After the removal of pupae, DSP (Lomant’s reagent) fixed slides were made, which show the morphology of the fly (Figure 2). Slides were made that show the morphology of the fly with the anterior part and projections of the posterior wings (Figure 2). The patient was followed up after one week, and there were no signs of blepharitis or previous inflammation on the slit lamp, and no new pupa was seen.

## Discussion

Different species of flies have been identified as responsible for ophthalmomyiasis, like the housefly (Musca domestica), the Oestrus ovis (sheep botfly), the latrine fly (Fannia), and the cattle botfly (hypoderma) [8]. Among these, Oestrus ovis is the commonest. Ovis ophthalmomyiasis was first described in 1947 [9]. Myiasis is found to be rare in humans. It is frequently seen in areas of poor hygiene and at the location of sheep and goat husbandries [10]. Very rarely, ophthalmomyiasis externa can be caused by Dermatobia hominis with eyelid and conjunctival involvement [8]. The majority of cases reported belong to Middle Eastern countries [11]. This condition most commonly occurs in the spring and summer. Warm summer conditions are generally optimum for the development of houseflies, and it can be completed in a short period of seven to ten days; however, it may take even two months in suboptimal conditions [9]. In the present case, pupae of Musca domestica are seen macroscopically as well as microscopically (Figures 1-2).

Ophthalmomyiasis externa may present clinically with symptoms of conjunctivitis [12,13], blepharoconjunctivitis, punctate keratitis, and keratouveitis [14]. The present case had blepharoconjunctivitis initially, for which topical treatment was given by the general practitioner, so the conjunctivitis component was absent when she was presented. As larvae were removed at home with cotton buds, on slit lamp examination, no motile larvae were identified. Still, numerous glistening golden-brown pupae were seen on lashes adhered to the lid margin. A detailed slit-lamp examination should be done in patients with these blepharoconjunctivitis, especially in areas of slums with thick populations. Few reports in the literature show the presence of keratitis along with external ophthalmomyiasis [14]. So, other symptoms should also be given importance, and the treatment should be modified accordingly.

In such cases, mechanical removal of the larvae is one of the important points to be considered. As larvae are highly mobile, they can easily escape notice, and there is no improvement in the symptoms observed. Reports showed that topical instillation of 1% cyclopentolate 10 minutes prior and topical anesthetic drops were used to reduce larval motility [15]. These two drugs help in their removal and decrease the chances of missing the detection of larvae. In the present case, there were no motile larvae, so there was no need to put on cyclopentolate eye drops. In earlier reports in the literature, there was a use of topical 4% cocaine hydrochloride treatment because of its anticholinergic properties, but to the best of our knowledge, another drug used was cyclopentolate in similar cases [15]. Systemic ivermectin is also used to treat cases where nasal cavities are involved as a complication of ophthalmomyiasis externa. Ivermectin can also be administered topically [16]. Difficulty in mechanically removing these larvae is tenaciously adhering to conjunctiva and lid due to hook-like structures surrounding their head. Due to this adherence, forceps are used in the removal of larvae.

The use of topical antibiotics and steroid application after treatment to suppress inflammation and prevent secondary infections was reported in the literature, but as there was skin excoriation and ulceration around the lid margins, non-steroidal anti-inflammatory drugs (NSAIDs) eye drops and antibiotic ointment were preferred.

## Conclusions

In conclusion, external ophthalmomyiasis should be the differential diagnosis, especially in patients presenting with blepharoconjunctivitis, where itching is the main symptom. A detailed history should be taken, especially when the patient belongs to suburban and hot climatic places with thick populations, and a slit-lamp examination of the eye and adnexa, including the inner eyelids, should be carried out so as not to escape the diagnosis of a more serious condition called ophthalmomyiasis interna.
